# Interaction of Aspirin (Acetylsalicylic Acid) with Lipid Membranes

**DOI:** 10.1371/journal.pone.0034357

**Published:** 2012-04-17

**Authors:** Matthew A. Barrett, Songbo Zheng, Golnaz Roshankar, Richard J. Alsop, Randy K. R. Belanger, Chris Huynh, Norbert Kučerka, Maikel C. Rheinstädter

**Affiliations:** 1 Department of Physics and Astronomy, McMaster University, Hamilton, Ontario, Canada; 2 Canadian Neutron Beam Centre, National Research Council Canada, Chalk River, Ontario, Canada; Nagoya University, Japan

## Abstract

We studied the interaction of Aspirin (acetylsalicylic acid) with lipid membranes using x-ray diffraction for bilayers containing up to 50 mol% of aspirin. From 2D x-ray intensity maps that cover large areas of reciprocal space we determined the position of the ASA molecules in the phospholipid bilayers and the molecular arrangement of the molecules in the plane of the membranes. We present direct experimental evidence that ASA molecules participate in saturated lipid bilayers of DMPC (1,2-dimyristoyl-sn-glycero-3-phosphocholine) and preferably reside in the head group region of the membrane. Up to 50 mol% ASA molecules can be dissolved in this type of bilayer before the lateral membrane organization is disturbed and the membranes are found to form an ordered, 2D crystal-like structure. Furthermore, ASA and cholesterol were found to co-exist in saturated lipid bilayers, with the ASA molecules residing in the head group region and the cholesterol molecules participating in the hydrophobic membrane core.

## Introduction

The molecular mechanism by which drugs interact with cell membranes has become a central issue in pharmacological science [1]. Aspirin (acetylsalicylic acid, ASA) is one of the most commonly used analgesic drugs. From infrared spectroscopy Casal, Martin and Mantsch speculated decades ago that aspirin is located in the lipid head group region of phospholipid bilayers [2]. We used x-ray diffraction to study the interaction between Acetylsalicylic Acid and saturated phospholipid bilayers made of dimyristoylphosphocholine (DMPC). Membranes containing up to 50mol% ASA were prepared.

In-plane and out-of-plane structure of the membranes in their gel (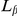) phase were determined from 2D x-ray intensity maps covering large areas of reciprocal space. We determine the location of the ASA molecules in the bilayer from electron density profiles perpendicular to the membranes and present the first direct experimental proof that ASA molecules participate in lipid bilayers and are located in the head group region of the bilayers. From wide angle x-ray diffraction experiments the arrangement of lipid and ASA molecules in the plane of the membrane was determined. While in pure DMPC bilayers, lipid head groups and tails show a high degree of positional order, small amounts of ASA lead to a disordered, fluid-like membrane. A 1∶1 (lipid:ASA) ratio was found to be the solubility limit of ASA molecules in saturated phospholipid bilayers, with one ASA molecule attached to each lipid head group. We also investigated a membrane containing 5mol% ASA and 15mol% cholesterol and observed that ASA and cholesterol molecules coexist in saturated lipid membranes.

## Materials and Methods

### Sample preparation

**Figure 1:**
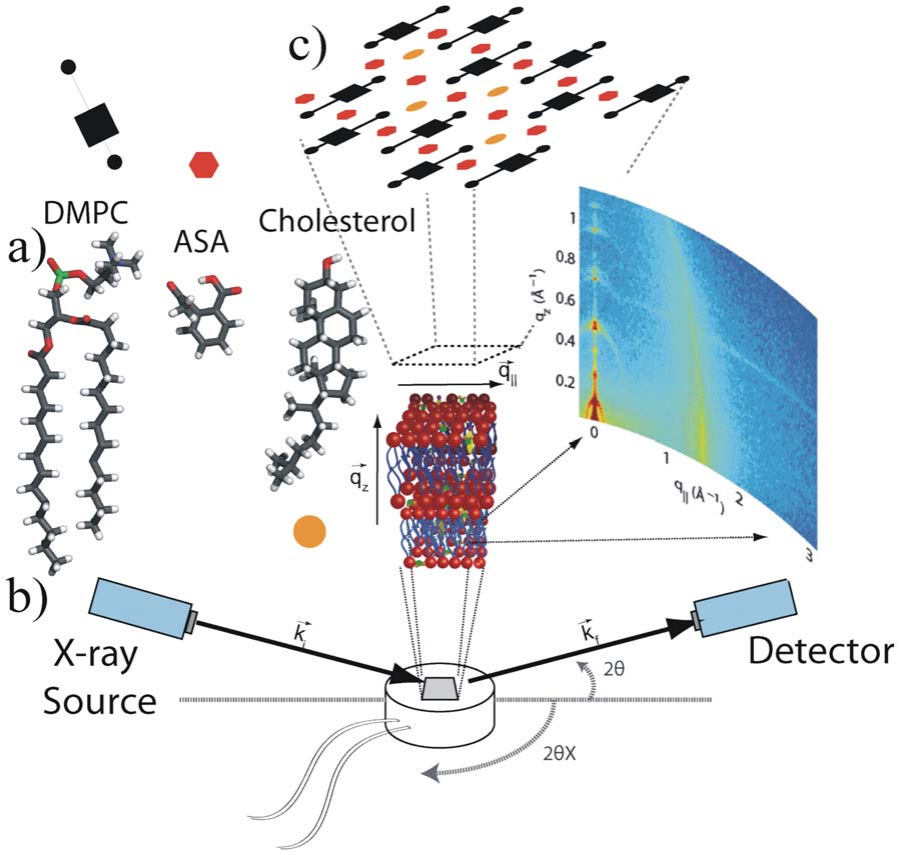
Experimental overview. a) Lipid, ASA and cholesterol molecule.b) Schematic diagram of the x-ray scattering experiment. The in-plane and out-of-plane structure of the membranes can be determined from the 2D intensity maps. Highly oriented multi lamellar membranes are used. 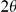 and 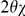 are the out-of-plane respective in-plane diffraction angles. c) Top view of the membrane to illustrate the molecular arrangement in the plane of the membrane. Lipids are depicted by a head group (▪) and two tails (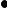). ASA and cholesterol molecules are represented by a hexagon and a circle, respectively.

Highly oriented multi lamellar membranes were prepared on single-side polished silicon wafers. 100 mm diameter, 300 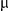
m thick silicon (100) wafers were pre-cut into 2
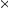
2 cm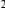 chips. 1,2-dimyristoyl-sn-glycero-3-phosphocholine (DMPC), acetylsalicylic acid (ASA) and cholesterol (depicted in Figure 1 a)) were mixed in different ratios and dissolved in a 1∶1 chloroform/2,2,2-trifluoroethanol (TFE) solution at a concentration of 15 mg/mL. The lipid solution did not spread well on ultrasonic-cleaned wafers and de-wetted during drying. The silicon substrates were, therefore, cleaned in a piranha acid solution made of 98% concentrated H
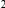
SO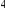 and 30% concentrated H
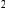
O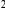 at a ratio of 3∶1 by volume. Wafers were placed in this solution, covered with parafilm and heated to 298 K for 30 minutes. This treatment removes all organic contamination and leaves the substrates in a hydrophilic state. We used silanization to cover the silicon surface through self-assembly with organo functional alkoxysilane molecules (APTES). The organic part of the APTES molecules was found to provide a perfect hydrophobic interface for the formation of the biological tissue. A 1% (by volume) solution of APTES and 99% ethanol was prepared. The wafers were immersed in the APTES solution and covered with parafilm, heated to 298 K and placed on a tilting incubator (20 speed, 3 tilt) for 12 hours. The tilting incubator creates a circular flow in the beaker to ensure an even APTES distribution and prevent buildup on the surface of the wafers. The wafers were then placed in a clean pyrex dish and annealed in vacuum at 388 K for 3 hours to create a uniform coverage of the APTES molecules on the surface [3]. Each wafer was thoroughly rinsed three times by alternating with 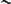
50 mL of ultrapure water and methanol. The methanol was cleaned using a 0.2 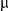
m filter before use to avoid surface contamination. The tilting incubator was heated to 313 K and the lipid solution was placed inside to equilibrate. The wafers were rinsed in methanol, dried with nitrogen gas and placed in the incubator. 200 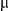
L of lipid solution was applied on each wafer, and the wafers covered with a petri dish to let the solvent evaporate slowly to allow time for the membranes to form. Wafers were tilted during the drying process for 30 minutes (speed 15, tilt 1) such that the lipid solution spread evenly on the wafers. After drying, the samples were placed in vacuum at 313 K for 12 hours to remove all traces of the solvent. The bilayers were annealed and rehydrated before use in a saturated K
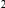
SO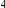 solution which provides 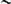
98% relative humidity (RH). The hydration container was allowed to equilibrate at 293 K in an incubator. The temperature of the incubator was then increased gradually from 293 K to 303 K over a period of 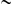
5 hours to slowly anneal the multi lamellar structure. This procedure results in highly oriented multi lamellar membrane stacks and a uniform coverage of the silicon substrates. About 3,000 highly oriented stacked membranes with a thickness of 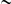
10 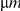 are produced using this protocol. The samples were stored in a refrigerator at 5

C and heated to 55

C for 1 h before scanning to erase a possible thermal history. This procedure in particular destroys possible crystalline 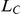 or sub-gel phases that may form during storage at low temperatures and low hydration, as has been reported in [4]. The high sample quality and high degree of order is necessary to determine in-plane and out-of-plane structure of the membranes and the position of the ASA molecules with high spatial resolution. Table 1 lists all samples prepared for this study.

**Table 1:** List of samples used.

Sample	DMPC (mol%)	ASA (mol%)	cholesterol (mol%)	Unit Cell	area per lipid (Å 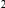 )	lipid tilt (deg)	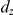 -spacing (Å)
1	100	0	0	head-groups: 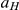 = 8.773 Å, 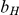 = 9.311 Å, 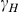 = 90  , lipid tails: 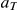 = 4.966 Å, 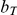 = 8.247 Å, 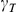 = 94.18 	40.84 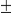 0.1	6.5	55.3
2	99	1	0	lipid tails: 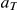 = 4.23 Å, 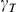 = 120 	41.4 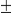 0.7	7.4	53.6
3	95	5	0	lipid tails: 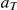 = 4.21 Å, 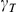 = 120 	41.0 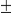 0.7	8.0	55.3
4	90	10	0	lipid tails: 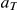 = 4.22 Å, 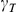 = 120 	41.1 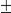 0.7	6.8	55.2
5	85	15	0	lipid tails: 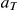 = 4.21 Å, 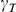 = 120 	40.9 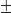 0.7	5.0	49.1
6	80	20	0	head-groups: 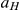 = 8.742 Å, 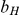 = 9.308 Å, 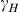 = 90  , lipid tails: 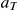 = 4.15 Å, 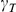 = 120 	39.8 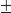 0.1	6.7	46.7
7	75	25	0	lipid tails: 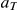 = 4.22 Å, 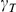 = 120 	41.2 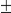 1.0	5.6	49.1
8	70	30	0	lipid tails: 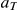 = 4.25 Å, 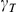 = 120 	41.7 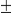 1.2	6.3	49.1
9	60	40	0	lipid tails: 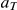 = 4.19 Å, 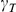 = 120 	40.5 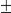 0.7	5.4	49.2
10	50	50	0	head-groups: 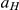 = 8.729 Å, 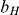 = 9.337 Å, 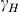 = 90  , lipid tails: 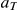 = 4.950 Å, 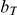 = 8.252 Å, 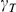 = 93.80  , ASA: 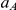 = 5.74 Å, 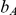 = 3.30 Å, 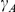 = 90 	40.75 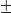 0.1	6.7	55.6
11	80	5	15	lipid tails: 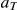 = 4.23 Å, 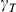 = 120 	41.3 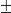 0.9	3.5	49.1

List of all the samples prepared for this study, and their molecular composition. Unit cell dimensions, areas per lipid, lipid tilt angles, and 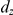 spacings are also given. See text for details.

### X-ray scattering experiment

Out-of-plane and in-plane x-ray scattering data was obtained using the Biological Large Angle Diffraction Experiment (BLADE) in the Laboratory for Membrane and Protein Dynamics at McMaster University. BLADE uses a 9 kW (45 kV, 200 mA) CuK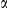 rotating anode at a wavelength of 1.5418 Å. Both source and detector are mounted on movable arms such that the membranes stay horizontal during the measurements. Focussing multi-layer optics provides a high intensity parallel beam with monochromatic x-ray intensities up to 10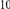 counts/(mm
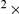
s). This beam geometry provides optimal illumination of the solid supported membrane samples to maximize the scattering signal. All data were obtained in grazing incidence, small and wide angle scattering geometry. A sketch of the scattering geometry is shown in Figure 1 b). By using highly oriented membrane stacks, the in-plane (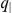) and out-of-plane (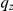) structure of the membranes can be determined. From the high resolution x-ray diffraction experiments we determine the molecular structure of the membranes in two different ways: (1) the out-of-plane membrane structure to determine the location of the different molecules in the membrane with sub-nanometer resolution and (2) the lateral organization of the different molecular components in the plane of the membrane, as sketched in Figure 1 c). The result of such an x-ray experiment is a 2D intensity map of a large area (0.03 Å
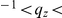
1.1 Å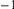 and 0 Å
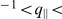
3.1 Å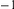) of the reciprocal space, as sketched in Figure 1 b). All scans were measured at 20

C and 50% hydration, in the gel (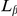) phase of the bilayers [5], [6]. Structural features are more pronounced in dry samples as fluctuations, which lead to attenuation and smearing of Bragg peaks, are strongly suppressed. The measurement of high-order Bragg peaks results in a high spatial resolution.

Specular reflectivity allows the determination of the structure and composition of membranes perpendicular to the plane of the membranes (see, e.g., [7], [8]). The intensity of the reflected beam as a function of the perpendicular momentum transfer, 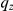, is given by:
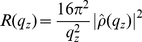
(1)

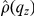
 is the one-dimensional Fourier transform of the electron density 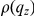, defined by:
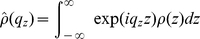
(2)Because of the stacking of the membranes, i.e., the convolution with the lamellar structure factor, the Fourier transform is not continuous but discrete. The different Fourier components are observed in the experiment as the integrated intensities of the out-of-plane Bragg peaks. 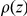 is approximated by a 1D Fourier analysis [9]:
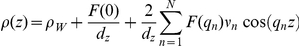


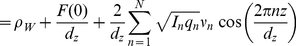
(3)

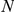
 is the highest order of the Bragg peaks observed in the experiment and 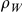 the electron density of bulk water. The integrated peak intensities, 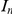, are multiplied by 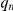 to receive the form factors, 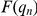 [10], [11]. The bilayer form factor 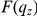, which is in general a complex quantity, is real-valued in the case of centro-symmetry. The phase problem of crystallography, therefore, simplifies to the sign problem 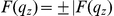, and the phases, 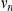, can only take the values 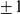. The phases 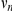 are needed to reconstruct the electron density profile from the scattering data following Equation (3). When the membrane form factor 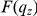 is measured at several 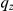 values, a continuous function, 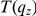, which is proportional to 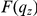, can be fitted to the data [10]–[13]:
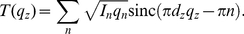
(4)Once an analytical expression for 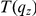 has been determined from fitting the experimental peak intensities, the phases 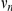 can be determined from 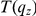 – this will be demonstrated below.

## Results

### In-plane structure

**Figure 2:**
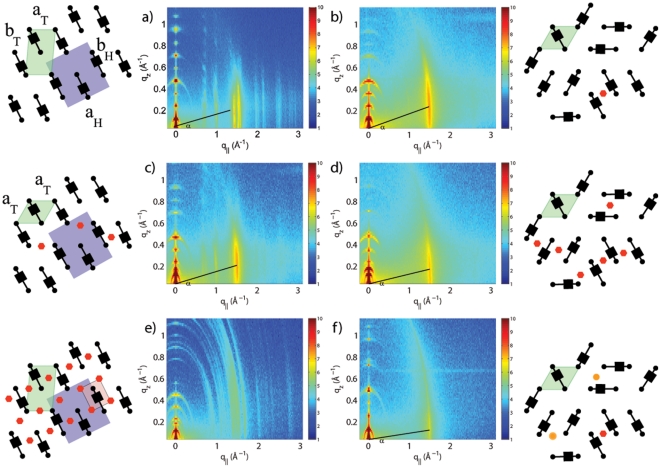
2D x-ray intensity maps. 2D x-ray intensity maps of Samples 1, 3, 6, 9, 10 and 11 (100% DMPC, 5mol% ASA, 20mol% ASA, 40mol% ASA, 50mol% ASA in DMPC and 5mol% ASA and 15mol% cholesterol in DMPC. The cartoons depict a top view of the in-plane structure (see Figure 1 c)) as determined from an analysis of the Bragg peaks along the in-plane axis, 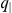.

Figure 2 displays 2D x-ray intensity maps for Samples 1, 3, 6, 9, 10 and 11. The arrangement of the different molecular components in the plane of the membranes can be determined from the the in-plane scattering along 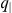. As introduced by Katsaras and Raghunathan [14], [15], different molecular components, such as lipid tails, lipid head groups and also ASA and cholesterol molecules, can form molecular sub-lattices in the plane of the membrane leading to non-overlapping sets of Bragg peaks.

The 100% DMPC sample (Sample 1) in Figure 2 a) shows a number of well developed in-plane Bragg peaks along the 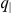-axis. The diffracted intensity has a distinct rod-like shape, typical for a 2D system. The out-of-plane scattering along 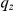 shows pronounced and equally spaced Bragg intensities due to the multi lamellar structure of the membrane sample. Analysis of the data and determination of the corresponding in-plane and out-of-plane structure will be discussed in detail below.

Some qualitative conclusions can already be drawn from the 2D data. The pattern changes by addition of 5mol% ASA (Figure 2 b)): the in-plane scattering shows one pronounced feature, only. Fewer Bragg peaks point to a short-ranged ordered, more fluid-like structure. Bragg peaks and molecular order are observed again at 20mol% ASA in Figure 2 c). Higher concentrated samples, such as 40mol% in Figure 2 d) appear to be disordered until an ordered pattern is observed at a concentration of 50mol% ASA (Figure 2 e)). The sample that contains ASA and cholesterol (Figure 2 f)) shows the fingerprint of a disordered membrane. The data in Figure 2 cover a large area of reciprocal space and are important to develop the molecular structure of the membrane systems. They are in particular important to detect or exclude structural features with mixed in-plane and out-of-plane properties, such as molecular tilts, which would in scattering with mixed, 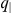 and 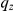 components. The cartoons next to the data in Figure 2 display the corresponding molecular structures, as determined from the analysis below.

**Figure 3:**
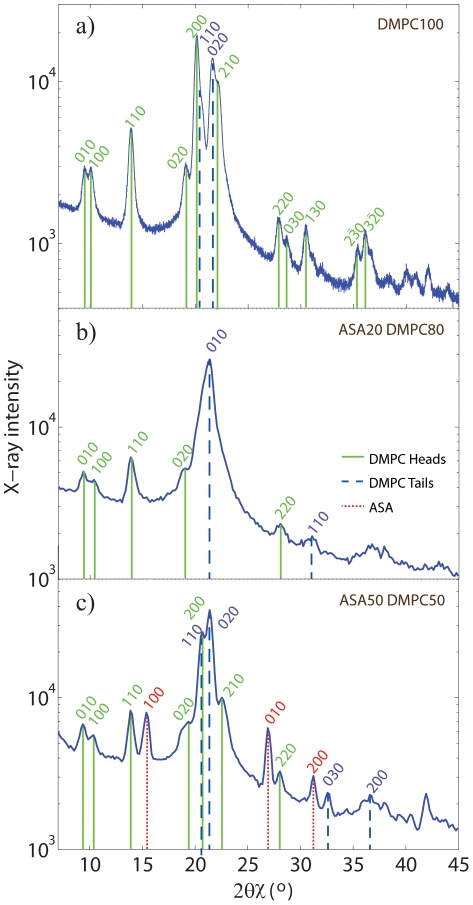
In-plane scattering integrations and fits. In-plane scattering of the Samples 1, 6 and 10. Slices 0.03 Å
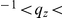
0.3 Å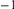 were integrated to increase the statistics and enhance the data quality. Bragg peaks assigned to lipid head groups are plotted in green, lipid tails in blue, and ASA in red. a) 100% DMPC: data are described by an orthorhombic unit cell for the head groups and a monoclinic tail unit cell. b) The 20mol% ASA sample shows an ordered head group lattice, and a disordered, hexagonal tail lattice. c) Ordered structures for head groups (orthorhombic), tails (monoclinic) and ASA molecules (orthorhombic) are found for 50mol% ASA.

To determine the in-plane structure, data were cut along the 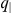-axis. Slices 0.03 Å
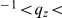
0.3 Å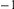 were integrated to enhance the data quality. The results for Sample 1 are shown in Figure 3 a). As depicted in the cartoon in Figure 2 a), the Bragg peaks were assigned to two different molecular lattices, the lipid head groups and the lipid tails. An orthorhombic head group lattice (planar space group p2) with lattice parameters 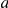 = 8.773 Å and 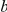 = 9.311 Å (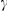 = 90) was found to best fit the data. The lattice of the lipid tails is commensurate with the head group lattice and the unit cell is determined by the relations [14], [15]:
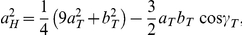


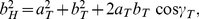
(5)

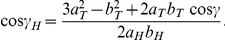
The subscripts 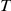 and 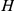 denote parameters of the tail and head group lattices, respectively. Solving these equation gives a monoclinic unit cell with parameters 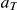 = 4.966 Å, 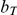 = 8.247 Å and 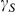 = 94.18. The positions of the corresponding Bragg peaks are superimposed with the data in Figure 3 a) and show an excellent agreement. The molecular structure is shown in the cartoon to Figure 2 a) and the two units cells are drawn. The orthorhombic unit cell of the head group lattice contains two lipid molecules and has an area of 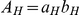 = 81.69 Å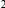. The area per lipid can also be determined from the unit cell of the tails, which contains one lipid molecule, to 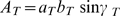 = 40.84 Å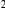.

The tilt of the lipid molecules in the gel phase can be determined from the scan in Figure 2 a). The diffracted intensity of the lipid tail peak at 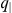 = 1.48 Å
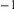
is not distributed homogenously along the rod, however peaks at a 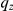 value of 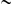
0.2 Å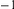, corresponding to a tilt angle of 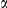 = 6.5 degs. Similar peak maxima are observed in the head group Bragg-rods, which leads to the conclusion that the whole lipid molecule is tilted by 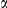.

Only one peak is observed at 5mol% ASA in Figure 2 b). Even at this relatively low concentration (ASA:lipids = 1∶20) the presence of the ASA molecules inhibits long-range order of the lipid head groups or tails as evidenced by the absence of Bragg peaks belonging to head or tail unit cells. From the data in Figure 2 it can be concluded that the lattices belonging to head groups and ASA molecules have a high degree of positional disorder. In the case of ASA this is most likely the result of a stochastic distribution of the ASA molecules in the bilayer. The area per lipid can be determined when assuming that the lipid tails form a densely packed structure with hexagonal symmetry (planar group p6). The lipid area can then be determined from the distance between two head groups respective two lipids tails on a hexagonal lattice to 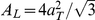. As the average distance between two tails is determined by the position, 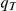, of the correlation peak to 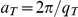, the area per lipid is determined to 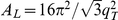 = 41.0 Å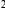. The corresponding unit cell parameters and areas per lipid are given in Table 1; the hexagonal unit cell is also drawn in the cartoon to Figure 2 b), where the molecular structure is sketched.

A pattern of Bragg peaks related to ordering of the lipid head groups is observed at an ASA concentration of 20mol% ASA in Figure 2 c); however only one peak related to the lipid tails is observed pointing to a positional disorder of the tails. The presence of the ASA molecules at a ratio of 1∶5 (ASA:lipid) induces a long-ranged ordered state between the lipid head groups, however, has little to no effect on the lipid tails. We find an orthorhombic unit cell for the head groups and a hexagonal symmetry for the lipid tails. The lattice parameters were determined from fitting the peak pattern in Figure 3 b) and are given in Table 1.

Higher concentrated samples (Samples 7–9) showed a disordered membrane structure with only the lipid correlation peak; 40mol% is shown as an example in Figure 3 d). The 20mol% sample therefore appeared to be special as this 1∶5 ratio between ASA and lipids lead to an ordering between the lipid head groups.

Several Bragg peaks are observed at the high ASA concentration of 50mol% in Figure 2 e). The Bragg peaks can be assigned to the ordering of the lipid tails and the lipid head groups. We find additional peaks in the pattern in Figure 3 c) that we assign to ordering of the ASA molecules. Orthorhombic unit cells for head groups and ASA molecules, and a monoclinic cell for the lipid tails were determined. In this structure, each lipid molecule “hosts” one ASA molecule. While 40mol% ASA can still be dissolved in the DMPC bilayer, 50mol% ASA leads to a non-physiological, highly ordered state, which clearly marks the solubility limit of ASA in saturated phospholipid bilayers. The unit cell dimensions, lipid areas and lipid tilt for all samples are given in Table 1; the corresponding molecular structures and unit cells are sketched in the cartoons in Figure 2.

### Out-of-plane structure and electron densities

**Figure 4:**
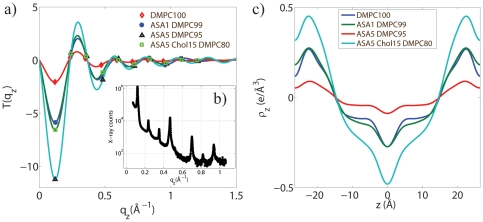
Electron density profile process. 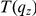
 (a) and calculated relative electron density profiles 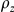 (c) for 100% DMPC, 1mol% ASA, 5mol% ASA, and 5mol% ASA 15mol% cholesterol. The phases, 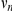, are determined by the sign of 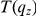 at the particular 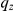-value. b) Out-of-plane scattering of the 5mol% ASA sample as an example. Seven pronounced Bragg peaks are visible.

The 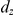-spacing between two neighboring membranes in the stack can be determined from the distance between the well developed Bragg reflections (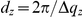) along the out-of-plane axis, 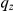. The integrated intensity of these peaks is used to calculate the electron density profile perpendicular to the bilayers following Equation (3). Figure 4 b) shows typical out-of-plane data taking Sample 3 containing 5mol% ASA as an example. Seven pronounced Bragg peaks are observed. The 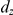 for this sample was determined to be 55.3 Å. Out-of-plane scans were measured for all samples in order to calculate the electron density profiles.

Figures 4 a) and c) display 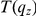 and relative electron densities as determined using Equations (4) and (3) for Samples 1, 2, 3, and 11 (100 mol% DMPC, 1 mol% ASA, 5 mol% ASA, and 5 mol% ASA with 15 mol% cholesterol). Up to 10 Bragg orders were observed. 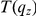 was fit to the experimentally determined peak intensities using Equation (4) to determine an array of phases 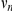 out of the corresponding 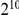 combinations of 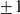. Figure 4 b) shows the best fits for all samples. All samples were well fit by the phase array 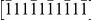. The corresponding relative electron densities in Figure 4b) show well developed features, which allow the determination of molecular positions in the membranes, as will be discussed below.

## Discussion

The in-plane structure of the multi-component membranes was determined from the 2D measurements in Figure 2 and the analysis in Figure 3. Ordering of the molecular sub-lattices of lipid head groups, tails, and ASA molecules was observed by analyzing the corresponding sets of Bragg peaks. While the DMPC lipids showed ordered head group and tail structures in the pure lipid membranes, small amounts of ASA were found to lead to a suppression of long-range order and a more fluid-like structure of the bilayers. 50mol% ASA was determined to be the solubility limit of ASA in saturated lipid bilayers resulting in a non-physiological 2D crystal-like state. At this concentration, each lipid molecule “hosts” one ASA molecule. As a special case, addition of 20mol% ASA (a 1∶5 ratio between ASA and lipid molecules) resulted in an ordered state of the head groups, as observed in the in-plane structure in Figure 2 c), while the tails still showed a fluid-like state with a high degree of positional disorder. This observation lead us to conclude that the ASA molecules preferably interact with lipid head groups and may, therefore, be located in the lipid head group region.

Lipid areas for all samples were determined from the in-plane scattering (see Table 1). The area that we determine for the pure DMPC sample can be compared to results published by Tristram-Nagle, Liu, Legleiter and Nagle [9], who provided a reference for the structure of gel phase DMPC membranes. The authors find an area per lipid of 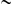
47 Å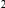 in fully hydrated bilayers at T = 10

C. The membranes in our study were measured at T = 20

C, however, significantly de-hydrated to 50% RH to enhance structural features. We note that the x-ray scans in Figure 2 show significantly more features as compared to the data observed by Tristram-Nagle *et al.* We determine lipid areas of 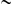
41 Å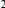. De-hydration obviously leads to a more closely packed lipid structure. An interesting observation is the fact that the area per lipid is almost constant for all ASA concentrations. It seems that the ASA molecules fill existing voids in the head group structure and do not increase the area per lipid (within the resolution of this experiment). This observation may be relevant to understand the physiological functioning of ASA. Changing the area per lipid changes important material properties of the membranes, such as permeability and elasticity. Future experiments will, therefore, determine structure of membranes containing ASA in fully hydrated gel and fluid phase membranes to elucidate this point in physiologically more relevant systems.

**Figure 5:**
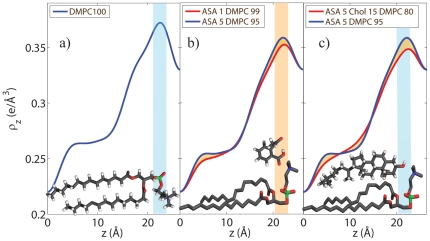
Normalized electron density profiles. a) Electron density profile of Sample 1 (100% DMPC) . b) The position of the ASA molecule can be determined from the electron densities of Sample 2 (1mol% ASA) and Sample 3 (5mol% ASA). The ASA molecule can be placed in the head group region of the bilayer, at 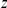 values of 16 Å
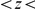
21 Å, with the hydrophilic oxygen groups at a 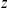 position of 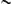
21 Å, pointing towards the hydration water. c) By comparing electron densities of Sample 3 and Sample 11 the position of the cholesterol molecule can be determined. Cholesterol and ASA molecules co-exist in saturated lipid bilayers and cholesterol most likely takes an upright position with the hydrophilic head pointing towards the aqueous environment. DMPC, ASA and cholesterol molecules are drawn to visualize the most likely positions and orientations.

The position of the ASA molecule in the bilayer can be determined from electron density profiles. 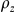 of pure DMPC (Sample 1), 1mol% ASA (Sample 2), 5mol% ASA (Sample 3), and 5mol% ASA/15mol% cholesterol (Sample 11) are shown in Figure 4. In order to put 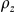 on an absolute scale, the electron densities were scaled to fulfil the condition 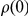 = 0.22 e/Å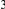 (the electron density of a CH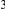 group) in the center of the bilayer, and 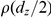 = 0.33 e/Å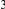 (the electron density of water, 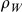) outside the bilayers. The electron density profile for DMPC (Sample 1) is depicted in Figure 5 a). The profile corresponds to a DMPC molecule in the well ordered gel state with both chains in all-trans configuration, as has been reported previously from [9]. The electron rich phosphorous group in the head group region can be identified by the peak in the electron density at 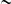
22 Å. 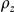 monotonically decreases towards the bilayer center at 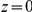; only CH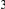 groups are found in the center.

To determine the position of ASA and cholesterol molecules in the bilayers, the electron densities of samples of different composition were compared. The electron densities for membranes containing 1mol% ASA and 5mol% ASA are depicted in Figure 5 b). Because both samples show a disordered scattering pattern, differences in the two electron densities should be directly related to the increased ASA content. The electron density in the 5mol% ASA was found to be increased in the head group region at 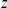 values of 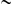
21 Å. As depicted in the Figure, an ASA molecule can be fitted at 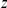 values of 16 Å
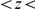
21 Å, with the hydrophilic, electron-richer oxygen groups at a 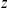 position of 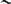
21 Å, pointing towards the hydration water. This orientation “protects” the hydrophobic part of the ASA molecule from the aqueous environment. The lower electron density towards the center of the bilayer most likely points to an increased disorder of the lipid tails in the 5mol% ASA sample, i.e., a higher number of gauche defects in the hydrocarbon chains. This conclusion is supported by the observation of a larger tilt angle of the lipid tails in the experiments, as listed in Table 1. The effect is qualitatively also visible in the 2D scans in Figure 2 a) and b), where the addition of 1mol% ASA suppresses long-range order of lipid head groups and tails and results in a disordered, more fluid state of the bilayers with a small 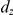 spacing.

A membrane containing ASA and cholesterol was included in the series (Sample 11) to study the possible interaction between the two molecules. The electron densities of Samples 3 (5mol% ASA) and 11 (5mol% ASA and 15mol% cholesterol) are shown in Figure 5 c). From the 2D scan in Figure 2 f) it can be concluded that this mixture still forms a homogenous multi lamellar structure and that cholesterol and ASA molecules co-exist in saturated lipid bilayers. By comparing the electron densities of Sample 3 and Sample 11 the position of the cholesterol molecule can be estimated. The electron density in the head group region is found to be lower when cholesterol is present as additional cholesterol acts as a spacer in chain region which dilutes the densities in head group region. The cholesterol ring structures lead to a slight increase in 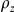 at 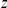 values of 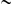
12 Å, while the electron density in the cholesterol sample starts to be lower below 8 Å as it has only one tail, as compared to two DMPC tails. We conclude that cholesterol takes an upright position also in the presence of ASA molecules, with the hydrophilic head pointing towards the aqueous environment. This upright position and orientation has been reported previously for cholesterol in saturated phospholipid bilayers made of DMPC and DPPC. The electron densities in Figure 5 c) definitely exclude that the cholesterol molecules take a flat position between the two leaflets, as it was reported recently for highly unsaturated lipid bilayers [16], [17]. While the cholesterol takes an upright position in the hydrophobic membrane core parallel to the lipid tails, the ASA molecules preferably reside in the head group region. Due to their position in the lipid membrane, the two molecules can be expected to have a different impact on membrane properties: while cholesterol is known to reduce permeability and increase membrane rigidity, ASA may enhance permeability and make membranes more fluid and flexible.

In summary, we determined the in-plane and out-of-plane structure of highly oriented, solid supported membranes containing up to 50mol% of Aspirin (acetylsalicylic acid, ASA) using x-ray diffraction. All membranes were in a low-hydration (50% RH) gel phase to enhance structural features in the scattering experiment. We present direct experimental proof that the ASA molecules participate in saturated phospholipid membranes. The molecules were found to reside in the lipid head group region. The presence of the ASA molecule has a distinct effect on the in-plane structure of the membranes: while pure DMPC bilayers form highly ordered head group and tail lattices, addition of 1mol% ASA suppresses long-range order and results in a disordered, fluid-like state. The maximum solubility of ASA in saturated lipid membranes was found to be 50mol%, which results in a structure where each lipid molecule “hosts” one ASA molecule. At an ASA/lipid ratio of 1∶5 (20mol% ASA) we observe positional order between the lipid head groups, while the lipid tails are still in a fluid-like state.

ASA and cholesterol molecules were found to co-exist in saturated lipid bilayers. As in saturated bilayers without ASA, cholesterol was determined to take an upright position. Our findings may be relevant to better understand the physiological function of Aspirin on a molecular level and for instance develop molecular models for the so-called “low-dose aspirin therapy”. The technique presented in this paper can in the future also be used to study the interaction of highly topical drugs with artificial membranes mimicking certain types of tissue, such as brain or muscle tissue.
